# Acute Stress in Health Workers in Colombia 2017–2021: A Cross-Sectional Study

**DOI:** 10.3389/ijph.2023.1606274

**Published:** 2023-09-01

**Authors:** Mery Gonzalez Delgado, Jesus David Cortes Gil, Deysy Lisette Rodriguez Araujo, Jose Joaquin Mira Solves, Erika Bibiana Rodriguez Gallo, Alejandra Salcedo Monsalve, Luz Angela Arrieta Arteta, Carolina del Pilar Villalba Toquica, Juan Carlos Morales Ruiz

**Affiliations:** ^1^ Facultad de Ciencias de la Salud y del Deporte, Especialización en Auditoría en Salud y Red Interprofesional Colombiana de Seguridad del Paciente, Fundación Universitaria del Área Andina, Bogotá, Colombia; ^2^ NOVA National School of Public Health, Public Health Research Center, Comprehensive Health Research Center, CHRC, NOVA University Lisbon, Lisbon, Portugal; ^3^ Facultad de Ciencias de la Salud y del Deporte, Especialización en Auditoría en Salud, Fundación Universitaria del Área Andina, Bogotá, Colombia; ^4^ Health Psychology Department, Miguel Hernández University, Elche, Spain; ^5^ Facultad de Ciencias de la Salud y del Deporte, Programa de Medicina, Fundación Universitaria del Área Andina, Bogotá, Colombia; ^6^ Oficina de Salud pública y Epidemiología, Clinica Universitaria Colombia, Bogota, Colombia; ^7^ Red Iberoamericana de Conocimiento en Seguridad del Paciente, Red Salud Colsubsidio, Bogotá, Colombia

**Keywords:** stress disorders, traumatic, acute, health personnel, psychosocial risk

## Abstract

**Objectives:** Analyze the presence of acute stress response after adverse events in human talent in Colombian health institutions from 2017 to 2021.

**Methods:** Cross-sectional study of prevalence, carried out on 838 members of the human talent in health (professionals, technicians, technologists, and auxiliaries) of Colombian health institutions in the study period with the application of the EASE instrument. Univariate analysis using descriptive statistical techniques, chi-square and Student’s t-test, and bivariate analysis with a Poisson regression model using the institucional SPSS v. 26.

**Results:** The prevalence of adverse events in the last 5 years was 33.8%, presenting levels of acute stress qualifying as Medium-high emotional overload at 21.91%, while extreme acute stress was at 3.53%. The prevalence of risk for presenting acute stress after being involved in an adverse event was PR: 1.30 (CI: 1.24–1.36).

**Conclusion:** Acute stress in human talent after adverse events is limiting health and care capacity and must be efficiently addressed by health institutions. Psychosocial risk is linked within the framework of the patient safety program and the institutional occupational health and safety management systems.

## Introduction

Patient safety is understood as the process by which the probability of harm to the patient during healthcare is sought to be reduced to a minimum acceptable level [1]; in this sense, in the Study of Prevalence of Adverse Effects in Latin American Hospitals (IBEAS) found a prevalence of 10% [2], and Ambulatory Care (AC) Services Across Latin America (LA) and the Caribbean (LAC) (AMBEAS/OPS) reported an adverse event prevalence of 5.2% [3, 4].

This is how the presentation of safety events related to healthcare unfolds: a patient suffers unpredictable and involuntary harm and requires support both physical and psychological; members of the human talent in health involved in the genesis of the event suffer negative impacts at a psychosocial, emotional, legal and professional level [5–9], and are designated second victims in a reputational crisis in the institution where the event occurred [10].

Therefore, among the negative impacts experienced by the second victim acute stress is one of the predominant symptoms that interferes with the proper performance of the professional and threatens the safety of patients [6, 11–13]. It is understood as a disorder related to a traumatic event such as patient safety events, which are related to direct contact with the event, which causes physical, psychological, and emotional effects [14]. Accordingly, psychosocial factors are understood as all those interactions that occur daily through work dynamics and in the work environment, and are present in all work environments regardless of the sector. In the health sector, the prevalence of acute stress due to exposure to psychosocial risk factors, after an adverse event, manifests itself as one of the conditions that produce the greatest impact on the health of health personnel, requiring effective intervention [6, 15–20].

However, health personnel worldwide during the period of this study also had to experience the effects on their mental health during the COVID-19 pandemic in the period from 2020 to 2021, where they were exposed to increased psychosocial strain and even greater probability of presentation of adverse events given the increase in patients and absenteeism of health personnel due to illness, among other causes, which materialized in disorders such as anxiety, depression, and acute stress as a consequence of work overload, repetitive exposure to traumatic events, and increased risk of contagion to them and/or their support network; this added to the stress resulting from the generation of adverse events, which is an aspect that must be taken into account in the study [21–24].

Due to the above, the present study aimed to analyze the presence of acute stress and presentation of the adverse event in human talent in Colombian health institutions in the period from 2017 to 2021.

## Methods

A cross-sectional study of healthcare providers in Colombia from 2017 to 2021 in 838 human talent in health (auxiliaries, technicians, technologists, and professionals) in health institutions in three Colombian regions (Caribbean, Andean, and Pacific) [25]. The study had the signing of informed consent and the approval of the Ethics Committee of the Fundación Universitaria del Área Andina, Fundación Universitaria Sanitas, and the Red Salud Colsubsidio. Participation was voluntary, and participants could stop participating at any time. The confidentiality of personal data was guaranteed.

### Instruments

The Acute Stress Scale (EASE) instrument was used and validated previously with a sample of health workers in Spain in primary care and hospitals [26, 27]. It was also employed in a study of four Latin American countries [20] (Table 1).

**TABLE 1:** Stress scale used among professionals caring Colombia 2017–2021.

Scale questions
Factor 1- affective responses
I keep my distance, resent dealing with people, and am irascible even at home
I have completely lost the taste for things that used to bring me peace of mind or wellbeing
I feel that I am neglecting many people who need my help
I cannot help but think of recent critical situations. I cannot get out of work
I have difficulty thinking and making decisions, have doubts, and have entered a kind of emotional blockage
Factor 2- fear and anxiety responses
I have difficulty empathizing with patients ‘suffering or connecting with their situation (emotional distancing and emotional anesthesia)
Worrying about not getting sick causes me a strain that is hard to bear
I am afraid I am going to infect my family
I feel on permanent alert. I believe my reactions now put other patients, colleagues, or myself at risk
I feel intense physiological reactions (shock, sweating, dizziness, shortness of breath, insomnia, etc.) related to the current crisis
Total (score ranges from 3 to 30) | Individual scores range from 0 to 3 (0 = it did not happen to me; 1 = It occurs to me on specific occasions; 2 = I happen often; 3 = I was like this continuously

Notes: Interpretability on the following categories based on score ranges were established: 0–9 points, good emotional adjustment; 10–14 points, emotional distress; 15–24 points, medium-high emotional overload; and ≥25 points, extreme acute stress.

The EASE scale was created to assess the possible acute stress from COVID-19 experienced during the COVID-19 pandemic in health sector personnel. The scale is self-applied through an application and has ten elements with four alternative responses each (Table 1).

The score scale ranges from 0 to 30 points and is structured into two factors; factor 1 refers to the emotional response, and factor 2 relates to the fears and anxiety experienced during the COVID-19 pandemic. The interpretability of the score is prioritized into four categories, good emotional adjustment (0–9 points), emotional distress (10–14 points), high emotional distress (15–24 points), and acute stress (>25 points).

In addition to the application of the instrument, a series of sociodemographic and work questions were included, in which the question “if he has been involved in an adverse event in the last 5 years” [sic] was included.

The sampling used was non-probabilistic sampling at convenience and was sent to the participants from different health institutions in the Andean, Pacific, and Caribbean regions through a Google form that contained the informed consent, a sociodemographic section, and variables of the work and the EASE instrument that was completed anonymously by the participants. The sample corresponds to health workers in health institutions of any level of complexity in the Andean, Caribbean, and Pacific regions. According to data from the National Policy of Human Talent in Health, in 2018 there were 599,890 health professionals; Casal’s [28] formula for infinite samples was employed, with a confidence level = 95%, a margin of error of 5%, meaning *n* = 384 to have a representative sample, and with the calculation of the non-response rate of 20%, the *n* = 461 participants, however, the answers received massively exceeded the calculated sample, with 834 health workers completing the questionnaire across three regions.

### Reliability

The scale was minimally adapted, with only a few words being changed to better encompass the Colombian study population. The metric attributes of the scale were evaluated using Cronbach’s and McDonald’s Omega Alpha coefficients; a value greater than .85 was considered acceptable for this analysis.

Internal consistency values were tested and considered as adequate >7 (Cronbach *α* = .874 and McDonald *ω* = .877); when all items of the EASE scale were considered.

### Statistical Analysis

A univariate analysis was fit considering the variables of interest—sex, marital status, educational level, working in more than one place, type of institution, and level of care indicate their percentage and size in the sample (Table 2).

**TABLE 2:** Characteristics sociodemographic and specific work of the sample (N = 838) in health institutions of three Colombian regions from 2017 to 2021.

Variable	Adverse event (2017–2021)				*p*-value
	No (*n* = 555)		Yes (*n* = 283)		
	n	%	n	%	
Sex					0.844
Man	115	20.7	57	20.1	
Women	440	79.3	226	79.9	
Civil Status					0.690
Married	174	31.4	103	36.4	
Divorced	23	4.1	12	4.2	
Single	225	40.5	109	38.5	
Free union	128	23.1	59	20.8	
Widow (er)	5	0.9		0.0	
Education level					0.064
Baccalaureate	5	0.9	1	0.4	
Postgraduate	159	28.6	97	34.3	
Undergraduate	156	28.1	90	31.8	
Technological or Technical	235	42.3	95	33.6	
Age in years	555	37.6*	283	37.8*	0.680
Seniority in the position	555	10.7*	283	7.3*	0.067
Years of experience in the health sector	555	13.1*	283	13.3*	0.068
Work in another institution					0.445
No	444	80.0	220	77.7	
Yes	111	20.0	63	22.3	
Type of institution where you work					0.491
Mixed	19	3.4	8	28	
Private	375	67.6	182	64.3	
Public	161	29.0	93	32.9	
Level of complexity of the institution					0.394
First level	142	25.0	66	23.3	
Second level	124	22.0	69	24.4	
Third level	139	25.0	60	21.2	
Fourth level	150	27.0	88	31.1	
Occupation					0.181
Professional nursing	123	22.0	78	28.0	
Nursing assistant	204	37.0	84	30.0	
General practitioner doctor	62	11.0	39	14.0	
Specialist doctor	77	14.0	36	13.0	
Others	89	16.0	46	15.0	
Service					0.014**
Pediatric and adult emergencies	138	24.9	57	20.1	
Hospitalization of various specialties (adults and pediatrics)	60	10.8	30	10.6	
Gynecology and Obstetrics	21	3.8	7	2.5	
Surgery	53	9.5	54	19.1	
Adult and pediatric intensive care unit	96	17.3	56	19.8	
External consultation	71	12.8	33	11.7	
Home care	9	1.6	4	1.4	
Pediatrics and neonatology	15	2.7	7	2.5	
Other services	92	16.6	35	12.4	

Notes: * Average for quantitative variables and percentage for qualitative variables. *p*-value with calculated test and Chi-squared. **p-value <0.05, *p-value <0.01.

Subsequently, a bivariate analysis was carried out between the same variables of interest and the acute stress scale in its four levels (good emotional adjustment, emotional distress, medium-high emotional overload, and extreme acute stress), using the Fisher’s exact test to have percentages of each category and to evaluate the possible statistical relationship (*p*-value <0.05) (Table 3).

**TABLE 3:** Bivariate analysis between the sample’s main variables (%) and the four stress levels of the Self-applied Acute Stress Scale (EASE) Colombia 2017–2021.

Variable	Estress acute level					
	n	Good emotional adjustment	Emotional distress	Medium-high emotional overload	Extreme acute stress	*p*-value
	838	*n* = 520	*n* = 168	*n* = 135	*n* = 15	
Sex						0.242
Men	172	22.7	18.5	15.6	13.3	
Women	666	77.3	81.6	84.4	86.7	
Marital status						0.648
Single	334	41.5	38.1	37.0	26.7	
Married or free union	464	54.2	56.6	56.3	73.3	
Divorced or widowed	40	4.2	5.4	6.7	0.0	
Education level						0.135
High school	6	0.8	1.2	0.0	0.0	
Postgraduate	256	31.0	33.9	27.4	6.7	
Undergraduate	246	29.0	32.1	28.2	20.0	
Technological or Technical	330	39.2	32.7	44.4	73.3	
Working in more than one place						0.083
No	664	79.2	81.6	74.1	100.0	
Yes	174	20.8	18.5	25.9	0.0	
Institution type						0.125
Mixed	27	4.2	2.4	0.7	0.0	
Private	557	66.5	61.9	69.6	86.7	
Public	254	29.2	35.7	29.6	13.3	
Level of attention						0.001*
First level	208	27.5	22.0	20.0	6.7	
Second level	193	22.1	28.0	22.2	6.7	
Third level	199	25.8	22.6	20.0	0.0	
Fourth level	235	24.6	27.4	37.8	86.7	
Occupation						
Nursing assistant	208	34.2	28.6	37.8	73.3	0.022**
Nurse	201	23.1	24.4	28.9	6.7	
Medical general practitioner	113	12.9	14.3	14.1	20.0	
Medical specialist	101	13.5	12.5	7.4	0.0	
Other	135	16.4	20.2	11.9	0.0	
Involved in adverse event						
No	555	78.3	61.9	54.1	33.3	0.001*
Yes	283	28.2	38.1	45.6	66.7	

Notes: **p-value <0.05, *p-value <0.01.

### Regression Analysis

A multiple regression analysis (Poisson) was performed between the stress score and the variables of interest already mentioned, generating prevalence ratios, confidence intervals, statistically significant *p*-values, and the overall model *p*-value. Variables with high collinearity were not included in the model.

The Variance Inflation Factors test (VIF mean) test was performed to evaluate possible collinearity between the model variables, considering a score of <2.5 as low multicollinearity.

## Results

### Characteristics of Sociodemographic and Specific Work of the Sample in Colombia From 2017 to 2021

A total of 838 health workers responded by applying all EASY scale items. As early results, we can mention that almost 80% were women, 34% were nursing assistants, and nearly 40% were married, and with a technical career as training, approximately 20% worked in more than one institution at the same time (Table 2). It was found that 66.4% work in private institutions and 30.3% in public institutions, and 28.4% in fourth-level institutions. Also reported was an average work experience of 13 years in the field, ranging from 7 to 47 (Table 2).

### Levels of the Self-Applied Acute Stress Scale

The bivariate analysis shows that extreme stress was reported more in women (86.7%), married or in a free union (73.3%), with technological or technical training (73.3%), working in private institutions (86.7%), at the fourth level of care (86.7%; *p*-value <0.01). A small number (3.53%; *p*-value < 0.01) were involved in an adverse event with a patient in the last 5 years, and the majority were nursing assistants (73.3%; *p*-value < 0.01) (Table 3).

**FIGURE 1:**
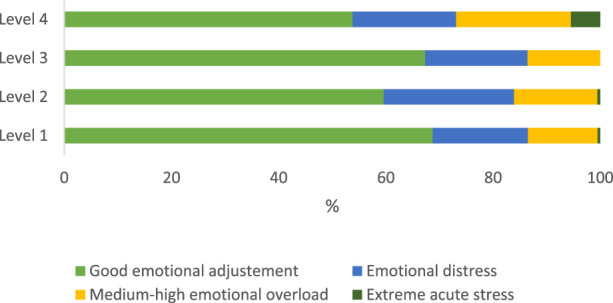
Percentage of emotional acute according to the level of attention of the working environment in Colombia from 2017 to 2021.

More detail in Figure 1 shows that the individuals with better emotional adjustment reported working in first- and third-level institutions. Those with worse emotional ajdustment reported having experienced extreme acute stress levels, and working in institutions of level 1.

**FIGURE 2:**
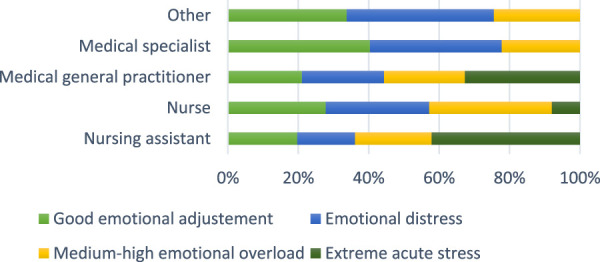
Percentage of emotional acute in the different levels according to the occupation Colombia 2017–2021.

Figure 2 shows the level of stress by occupation or position. Medical specialists reported higher percentages of good emotional adjustment and less stress, general practitioners and nursing assistants reported a higher percentage of having experienced extreme stress in Colombia from 2017 to 2021.

Multiple Poisson regression between the main characteristics of participants and the Acute Stress Scale score in Colombia from 2017 to 2021.

The multiple regression analysis allows us, after adjusting for other co-variables of the model, to identify that being a medical specialist (PR 0.81; *p*-value = 0.01; CI = 0.74–0.89) and working at the first attention level (PR 0.71; *p*-value = 0.01; CI 0.66–0.76) were protective factors for presenting high-stress levels during the pandemic (Table 4).

**TABLE 4:** Multiple Poisson regression between the main characteristics of participants and the Acute Stress Scale (EASE) score Colombia 2017–2021.

Acute stress	PR	*p*-value	CI 95%	
			LL	UL
Occupation				
Nurse	0.96	0.157	0.90	1.02
General practitioner	1.08	0.055	1.00	1.16
Medical specialist	0.81	0.000*	0.74	0.89
Other	0.96	0.312	0.90	1.04
Nursing auxiliary	1 ref			
Sex				
Woman	1.07	0.043**	1.00	1.14
Men	1 ref			
Marital status				
Married or free Union	1.05	0.078	1.00	1.10
Divorced or widowed	1.15	0.013**	1.03	1.28
Single	1 ref			
Level of institution				
Second level	1.18	0.000*	1.10	1.27
Third level	1.19	0.000*	1.10	1.28
Fourth level	1.41	0.000*	1.31	0.90
First level	1 ref			
Involved in a past event				
Yes	1.30	0.000*	1.24	1.36
No	1 ref			
Type of institution				
Private	1.49	0.000*	1.27	1.76
Public	1.64	0.000*	1.38	1.94
Mixed	1 ref			

Notes: LL, Lower Limit; UL, Upper Limit; *p-value <0.01; **p-value <0.05; Mean VIF collinearity 2.33; Model *p* < 0.01.

On the other hand, it showed that factors such as being a general physician (PR 1.08; *p*-value = 0.055; CI 1.00–1.16), being a woman (PR 1.07; *p*-value = 0.043; CI 1.00–1.14), being divorced or widowed (PR 1.15; *p*-value = 0.01; CI 1.03–1.28), having been involved in a stressful event previously (PR = 1.30; *p*-value = 0.01; CI 1.24–1.36), working in private institutions (PR = 1.49; *p*-value 0.01; CI 1.27–1.76), and working and public institutions (PR = 1.64; *p*-value = 0.01; CI 1.38–1.94), increased the chance of presenting higher levels of stress (Table 4)*.*

## Discussion

The present study showed important results as to how the prevalence of medium-high stress and acute stress was 21.91% and 3.53% respectively in the human talent in institutional health in the three Colombian regions, who reported adverse events in the study period. Equally, it revealed the high prevalence of medium and high stress in medical professionals (14.1%), nursing professionals (28.9%), and nursing assistants (37.8%), with mostly women responding (84.4%).

In this sense, a study that used the same instrument, the Self-applied Acute Stress Scale (EASE) tool, in countries in the Latin Americans (Argentina, Colombia, Chile, and Ecuador), on health providers during the COVID-19 pandemic, but in which it was not compared with the presentation of adverse events, found similar results, wherein medium-high stress had a prevalence of 27% [29]. However, studies are few that have measured the factors related to the presentation of acute stress after an adverse events that use this instrument in the region. Multiple studies have evaluated the prevalence of stress in health personnel in the world before and during the pandemic, which coincides with the high prevalence of stress in this population, being one of the factors associated, with employment dissatisfaction and belonging to occupations such as medicine and nursing [30]. Also, it is important to highlight that around the phenomenon of secondary victims, one of the main negative impacts is acute stress and post-traumatic stress that will coincide with what was found in this study [11, 13, 31, 32].

As a limitation, we can mention that we would have preferred more homogeneity in the professions of the sample, in general. The sample was predominated by nurses and nursing assistants, as well as being predominantly female due to the greater presence of that gender in the human talent in health [27]. The policy of human talent in Colombia health aims that the stratification is balanced and thus closer to the representability of the population of workers, which could not be achieved at all in the samples since participation was free, open, and voluntary.

Among its contributions, this study found the reaffirmation of the reliability of the (EASE) in health providers in the context of Colombia in consonance with the results of other studies made in Iberoamerica, like way measuring tool for acute effective stress in the health population, that before the presentation of an adverse event can be used in a routine way within the interventions protocols for secondary victims around the diagnosis of acute stress as impact-relevant in the phenomenon of secondary victims [26–29]. For another part, this study allows identifying some factors related to the prevalence of acute stress after the presentation of the adverse event in the study population of the country, being one of the first studies in this context.

For another part, due to the limitations of a non-probabilistic sample, the study cannot identify risk factors between the different health issues, ideally future studies should implementprobabilistic sampling and make epidemiological data designs that allow the estimation of risk factors associated with acute stress and the presentation of adverse events in the human talent in health that allow extrapolating the results.

In conclusion, the prevalence of acute stress following an event adverse result is relevant in the studied population, which demonstrates that it is necessary to prioritize diagnosis and secondary victims in health institutions Colombians, in the regions evaluated.

It is also suggested to implement intervention strategies within the framework of management and health and safety systems at work, within actions aimed at mitigating risk social psycho in health providers with professional and auxiliary nursing staff, because they are the largest population in the health institutions and more likely to present adverse events, for most activities related to patient care.

It is important to mention that this study served as an element for the elaboration of the National Guide of Recommendations for the Care of Secondary and Tertiary Victims after an Adverse Event in Colombian Health Institutions.
